# Case Series Study of Melioidosis, Colombia 

**DOI:** 10.3201/eid2508.170786

**Published:** 2019-08

**Authors:** José Y. Rodríguez, Soraya E. Morales-López, Gerson J. Rodríguez, Carlos A. Álvarez-Moreno, Kelin Esquea, Heidy Pinzon, Luis R. Ramirez, Lilian Moreno, Walter Ocampo, Martha L. Cepeda

**Affiliations:** Hospital Rosario Pumarejo de López, Valledupar, Colombia (J.Y. Rodríguez);; Centro de Investigaciones Microbiológicas del Cesar (CIMCE), Valledupar (J.Y. Rodríguez, G.J. Rodríguez);; Clínica Laura Daniela, Valledupar (J.Y. Rodríguez, K. Esquea);; Clínica Médicos LTDA, Valledupar (J.Y. Rodríguez, H. Pinzon);; Laboratorios Nancy Flórez García S.A.S., Valledupar (S.E. Moralez-López);; Universidad Popular del Cesar, Valledupar (S.E. Moralez-López);; Universidad Nacional de Colombia, Bogotá, Colombia (C.A. Álvarez-Moreno);; Clínica Universitaria Colombia, Clínicas Colsanitas, Bogotá (C.A. Álvarez-Moreno);; Grupo de Investigaciones Microbiológicas del Cesar, Valledupar (L.R. Ramirez, L. Moreno);; Corporación CorpoGen, Bogotá (W. Ocampo, M.L. Cepeda)

**Keywords:** melioidosis, Burkholderia pseudomallei, Colombia, pneumonia, bacteria

## Abstract

We report 7 cases of melioidosis in Colombia and comparision of 4 commercial systems for identifying *Burkholderia pseudomallei*. Phoenix systems were not a definitive method for identifying *B. pseudomallei*. For accurate identification, we recommend including this bacterium in the library databases of matrix-assisted laser desorption/ionization mass spectrometry systems in Latin America.

Melioidosis is an infectious disease caused by *Burkholderia pseudomallei*, a saprophytic soil bacterium (*1*). Recently, an increase in cases outside the Asia–Pacific region, including the Americas, has been reported. It is not clear whether this increased number of cases reflects an increase in incidence of this disease or improvements in its identification by microbiological laboratories and research facilities (*2*).

## The Study

We describe 7 cases of melioidosis in the Caribbean coast region of Colombia among patients who sought emergency services or were referred to 3 hospitals in the city of Valledupar (Figure). None of the patients reported travel abroad. Four patients were admitted during 2015 and 2016; we analyzed the corresponding isolates using 4 commercial methods and a molecular identification method. For the other 3 cases, which were diagnosed in 2014, we reviewed patient medical records and microbiological results. These isolates were not available. The study was approved by the ethics committees of Clinica Laura Daniela, Clínica Médicos, and Instituto Cardiovascular del Cesar.

**Figure:**
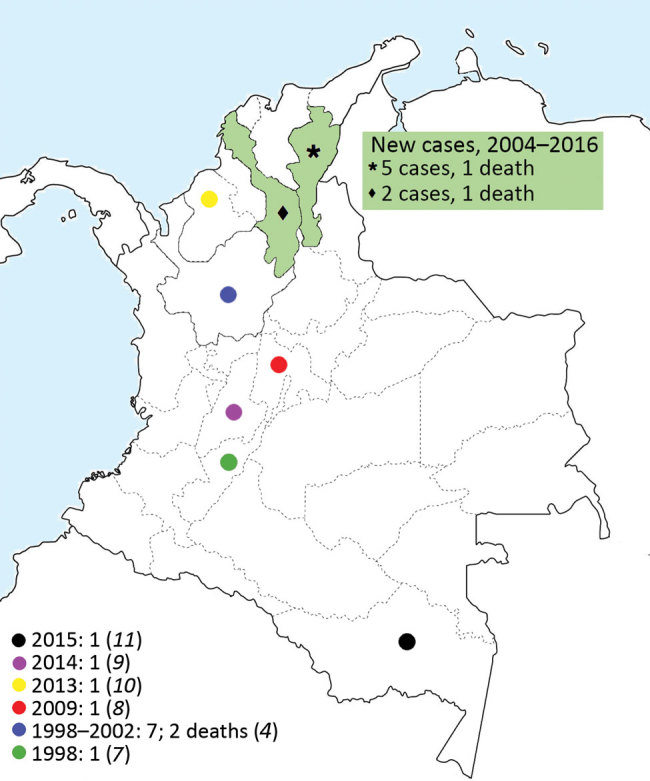
Geographic locations of 7 reported cases of melioidosis in Colombia.

Of the 7 patients, 6 (86%) were men; 2 patients were admitted to the intensive care unit (Table 1). The most common clinical presentation was bacteremic pneumonia (in 4 patients); 5 patients had a history of diabetes.

**Table 1:** Epidemiologic and demographic characteristics, underlying conditions, clinical manifestations and outcomes of patients with melioidosis in the Caribbean coast region of Colombia*

Patient no.	Patient age, y/ sex	Medical history and risk factors	Clinical manifestation	Type of sample	Empirical treatment	Appropriate treatment (duration)	ICU	Outcome	Relapse
1	72/M	Diabetes, arterial hypertension	Bacteremic pneumonia	Blood culture, urine culture, endotracheal aspirate	TZP + CLR	IP: MER + TMP/SMX (14 d); EP: NA	Yes	Died	NA
2	60/F	Diabetes, arterial hypertension, minor head injury with open scalp wound	Bacteremic SSTI	Wound secretion, blood culture	CFZ + CLI	IP: MER (10 d); EP: TMP/SMX (3 mo)	No	Alive	No
3	49/M	Diabetes, leprosy, erythema nodosum leprosum, chronic use of steroids	Bacteremic pneumonia	Blood culture, urine culture, sputum culture	TZP + CLR	IP: MER (10 d); EP: TMP/SMX (6 mo)	No	Alive	No
4	71/M	Arterial hypertension, multiple myeloma, bone marrow transplant, chronic use of steroids and thalidomide	Bacteremic pneumonia	Blood culture	TZP + DOX	IP: MER (14 d); EP: TMP/SMX (3 mo)	No	Alive	No
5	66/M	Diabetes, arterial hypertension	Bacteremic pneumonia	Blood culture, endotracheal aspirate	TZP	NA	Yes	Died	NA
6	56/M	Diabetes, chronic renal failure	UTI	Urine culture	TZP	IP: MER (10 d); EP: TMP/SMX (3 mo)	No	Alive	At 6 mo: bacteremia, UTI, SSTI
7	54/M	Tibia and fibula open fracture	SSTI	Wound secretion, blood culture	CFZ + CLI	IP: MER (10 d); EP: TMP/SMX (6 mo)	No	Alive	No

*CFZ, cefazolin; CLI, clindamycin; CLR, clarithromycin; DOX, doxycycline; EP, eradication phase; IP, intensive phase; MER, meropenem; NA, not available; SSTI, skin and soft tissue infection; TMP/SMX, trimethoprim/sulfamethoxazole; TZP, piperacillin/tazobactam; UTI, urinary tract infection.

The 4 strains isolated in 2015 and 2016 were identified as *B. pseudomallei* by Vitek Compact 2 (bioMérieux, https://www.biomerieux.com) and Microscan (Walkaway Beckman Coulter, https://www.beckmancoulter.com) but as *B. cepacia* by Phoenix (Becton Dickinson, http://www.bd.com) and as *B. thailandesis* by MALDI Biotyper v3.1 matrix-assisted laser desorption/ionization time-of-flight (MALDI-TOF) mass spectrometry (Bruker Daltonics, https://www.bruker.com). Internal transcribed spacer (ITS) DNA sequencing identified all the isolates as *B. pseudomallei*. Sequences of these 4 isolates were submitted to GenBank (accession nos. KX898558, KY659330, KY996759, and KY659331). According to patient medical records, the 3 isolates from 2014 were identified as *B. pseudomallei* using the Vitek or Microscan system or both (Table 2).

**Table 2:** Isolate dentification results by commercial systems, 16S rRNA sequencing analysis, and antimicrobial drug susceptibility of isolates of *Burkholderia pseudomallei* obtained from clinical specimens in the Caribbean coast region of Colombia*

Isolate no.	Commercial bacterial identification systems				ITS sequencing segments	GenBank accession no.			
	Vitek (% probability)	Walkaway/ Microscan (% probability)	Phoenix (% probability)	MALDI-TOF MS (score)			MIC, μg/mL		
							CAZ	MER	TMP/ SMX
1	ND	*B. pseudomallei* (93%)	ND	ND	ND	ND	2	<1	<2/38
2	*B. pseudomallei* (99%)	*B. pseudomallei* (99.5%)	ND	ND	ND	ND	>16	<1	<2/38
3	*B. pseudomallei* (94%)	*B. pseudomallei* (99.8%)	ND	ND	ND	ND	2	<1	<1/19
4	*B. pseudomallei* (94%)	*B. pseudomallei* (99.9%)	*B. cepacia* (99%)	*B. thailandensis* (1,899)	*B. pseudomallei*	KX898558	4	1	2/38
5	*B. pseudomallei* (94%)	*B. pseudomallei* (99.5%)	*B. cepacia* (99%)	*B. thailandensis* (1,898)	*B. pseudomallei*	KY659330	4	2	1/19
6	*B. pseudomallei* (95%)	*B. pseudomallei* (99.9%)	*B. cepacia* (99%)	*B. thailandensis* (1,898)	*B. pseudomallei*	KY996759	4	1	1/19
7	*B. pseudomallei* (94%)	*B. pseudomallei* (93%)	*B. cepacia* (99%)	*B. thailandensis* (1,898)	*B. pseudomallei*	KY659331	<2	2	1/19

*CAZ, ceftazidime; ITS, internal transcribed spacer; MALDI-TOF MS, matrix-assisted laser desorption/ionization time-of-flight mass spectrometry; MER, meropenem; ND, no data; TMP/SMX, trimethoprim/sulfamethoxazole.

MIC testing was performed using the system available in the institution where the patient was being treated: Microscan Walkaway (isolates 1, 2, 3, 5) and Phoenix systems (isolates 4, 6, 7). According to published cutoff points for *B. pseudomallei* (Clinical and Laboratory Standards Institute, https://clsi.org/standards/products/microbiology/documents/m45), all isolates were susceptible to trimethoprim/sulfamethoxazole and meropenem, and 2 isolates (isolates 2 and 7) were resistant to ceftazidime (MIC >16 μg/mL); however, isolate 7 was tested 2 more times using Microscan, resulting in a value of <2 μg/mL. Isolate 2 was not available for further analysis.

Within the genus *Burkholderia*, *B. pseudomallei*, *B. mallei*, and the *B. cepacia* complex are the species usually associated with human infection (*3**,**4*). Historically, melioidosis is recognized as a major cause of fatal pneumonia and sepsis in Southeast Asia, South Asia, and northern Australia. It is believed that movements of both humans and cargo have contributed to the dissemination. A phylogenetic reconstruction study determined the African origin of the American isolates and the overlapping of the introduction of *B. pseudomallei* into the New World with the height of the slave trade from Africa to the Americas (*5*).

In Colombia, melioidosis is not a disease of public health interest and thus could be underreported. However, Colombia is second to Brazil with the highest number of cases reported in South America (*1*). The actual number of cases is unknown, probably because of the lack of awareness and suspicion of the disease by health professionals, together with the absence of technology for proper diagnosis and the underreporting of diagnosed cases. At least 10 reported cases of melioidosis have been acquired in the Andean region of Colombia. Severiche published a report in 1998 about a patient with pneumonia (*6*). Montufar et al. described a series of 7 patients during 1998–2012, of whom 5 had bacteremic pneumonia (*7*). Since then, other cases of bacteremic pneumonia have been reported (*8*,*9*), as well as 1 case of chronic lung melioidosis in a patient on Colombia’s west Caribbean coast (*10*). Guzmán-Gómez et al. described a case of osteoarticular melioidosis acquired in the Colombian rainforest in 2015 (*11*), the only case confirmed by sequencing (ITS-16S rRNA gene). The areas in Colombia where melioidosis cases have been reported, including these new cases from the Caribbean coast, are consistent with the previously proposed model (*2*) (Figure). 

The isolation of *B. pseudomallei* from clinical specimens is the standard for a diagnosis of melioidosis. However, the microorganism is not often recovered and may not be correctly identified even when isolated. *B. pseudomallei* can be identified using commercial identification systems. However, these tests may fail to distinguish *B. pseudomallei* from *B. thailandensis* and other members of the *B. cepacia* complex (*12**,**13*). MALDI-TOF mass spectrometry is an accurate and rapid procedure for the identification of *B. pseudomallei* if the appropriate database is used (*14*). However, genotyping methods based on rRNA sequencing should be used for a more accurate diagnosis. We compared different phenotyping methods (Vitek, MicroScan Walkaway, Phoenix, and MALDI-TOF mass spectrometry) using 4 isolates from this report. Sequencing of the ITS region confirmed the identification of *B. pseudomallei* and matched the identifications obtained by the Vitek and Walkaway systems. However, the Phoenix system erroneously identified isolates as *B. cepacia*, and MALDI-TOF mass spectrometry incorrectly identified isolates as *B. thailandensis*. Although *B. pseudomallei* was included in the Phoenix database, we strongly recommend not using Phoenix as a single or final method to identify possible isolates of *B. pseudomallei* (for example, gram-negative, oxidase-positive, and positive-arginine bacilli). MALDI-TOF mass spectrometry is currently being introduced in Colombia, and we recommend the inclusion of *B. pseudomallei* in the database.

The treatment of melioidosis is prolonged and includes 2 phases: intensive treatment with intravenous antimicrobial therapy for 10–14 days using ceftazidime, imipenem, or meropenem; and an eradication phase with oral antimicrobial therapy for 3–6 months with trimethoprim/sulfamethoxazole alone or in combination with doxycycline (*15*). In the cases we report, treatment with meropenem was started, because it is easier to prescribe this antimicrobial drug in Colombia, given the high rate of extended-spectrum β-lactamase–producing *Enterobacteriaceae*; the use of ceftazidime is restricted for the same reason. Because of the severity of illness and the high rate of death from this disease, along with the required prolonged antimicrobial drug therapy and the small number of drugs available for its treatment, it is necessary not only to strengthen the public health surveillance and clinical suspicion of melioidosis but also to acquire tools that permit an adequate diagnosis, especially in potentially endemic areas, which, in the case of Colombia, could be an extensive geographic area.

## Conclusions

With the presence of *B. pseudomallei* on Colombia’s Caribbean coast confirmed, clinicians should suspect melioidosis in patients with risk factors, suggestive clinical symptoms, and microbiological isolates from clinical specimens of *B. pseudomallei* or other members of the *Burkholderia* genus. In this case, >1 microbiological identification method should be used, especially if the outdated Phoenix or MALDI-TOF mass spectrometry databases are used.

Finally, it is necessary to include melioidosis in a passive surveillance system, especially in those regions of Latin America where the environmental conditions create high probabilities for the presence of the disease. Currently, Colombia has a public health surveillance and control system under the supervision of the National Institute of Health, to which all health institutions must notify diseases of public health interest. To determine the true magnitude of melioidosis in Colombia, it must be included as a notifiable disease and measures established to enable its early diagnosis and treatment.
